# Mumps

**Published:** 1915-10

**Authors:** 


**Mumps.** L. J. Genella of New Orleans, in a letter to the Med. Council, Aug., alludes to a death reported in a previous issue and states that St. Bartholomew's Hospital reported a few deaths in 1913 and considers fatal cases as fairly common. These are usually regarded as due to meningo-encephalitis but he advances the theory that the toxins incapacitate the thyroid and reacts upon the choroid plexus so that the cerebro-spinal fluid is increased, with resulting rise of endocranial pressure.

